# The Effects of Septal Deviation, Concha Bullosa, and Their Combination on the Depth of Posterior Palatal Arch in Cone-Beam Computed Tomography

**Published:** 2016-03

**Authors:** Zahra Dalili Kajan, Jalil Khademi, Somayeh Nemati, Elmira Niksolat

**Affiliations:** 1 Dept. of Maxillofacial Radiology, School of Dentistry, Guilan University of Medical Sciences, Rasht, Iran.; 2 Dept. of Orthodontics, School of Dentistry, Guilan University of Medical Sciences, Rasht, Iran.; 3 School of Dentistry, Guilan University of Medical Sciences, Rasht, Iran.

**Keywords:** Hard Palate, Nasal Septum, Measurement, Cone-Beam Computed Tomography

## Abstract

**Statement of the Problem:**

Nasal breathing is the major pattern of air intake. Changes in breathing pattern alter the posture of the head, jaws and tongue that could change pressure on the jaw and teeth and affect their growth.

**Purpose:**

This study aimed to investigate the relationship between septal deviations (SD) per se and in combination with concha bullosa (CB) on maxilla; particularly the depth of palatal arch.

**Materials and Method:**

This descriptive-comparative study was performed on 116 cone-beam computed tomography (CBCT) images. The images were categorized into four groups (n=29) as follows; group 1: SD+CB, group 2: only SD, group 3: neither SD nor CB, and group 4: only CB. In coronal images, deviated septal length (DSL), angle of deviated septal curve (DSCA), palatal arch depth (PAD), palatal interalveolar length (PIL), PAD/PIL ratio, septal vertical length (SVL), maxillopalatal arch angle (MPAA), interjugum distance (IJD), and jugum angle (JA) were measured. The data were statistically analyzed with Tukey's HSD and Chi-square tests.

**Results:**

There were statistically significant differences in DSL and DSCA (*p*= 0.0001) among the four groups. The study groups were not statistically different regarding the IJD, JA, MPAA, PAD/PIL, PAD, PIL, and SVL. However, in group 1, PAD/PIL were significantly correlated with DSCA and DSL (*p*= 0.037, and *p*= 0.043, respectively).

**Conclusion:**

Based on the findings of this study, simultaneous occurrence of SD and CB influenced the depth and curve of the palatal bone. The PAD/PIL ratio was negatively correlated with the DSCA angle. This correlation was associated with a decrease in PAD, indicating that concurrent occurrence of SD and CB remarkably affected the palatal base of maxilla.

## Introduction

Nasal breathing is the primary mode of air intake that can be replaced by oral breathing due to obstructive or habitual causes.[1] Most common obstructive causes are hypertrophied adenoids, maxillary sinusitis, concha bullosa (CB), deviated nasal septum, and hypertrophied inferior concha, respectively.[2]

The nasal septum is located in the medial wall of the nasal cavity. It extends from the roof to the floor of the nasal cavity. It lies between the cribriform plate and the hard palate superio-inferiorly. It also spreads from the septal cartilage anteriorly to the vomer and the perpendicular plate of the ethmoid bone posteriorly. Three projections in variable sizes called the inferior, middle, and superior nasal conchae form the lateral wall of the nasal cavity. Pneumatization of the concha, called concha bullosa (CB), is one of the most common variations of sinonasal structures. CB is most frequently found in the middle concha,[3] and in association with septal deviations (SD). A sort of relationship is defined between the CB and SD.[4-7]

In the mouth breather patients, various jaw deformities such as narrow maxillary arch, posterior crossbite, more overjet, and a deep and dome-shaped palate are detected.[8] A deep and dome-shaped palate can induce stress on the nasal septum and cause septal deviation.[9]

The effects of oral respiration from nasal blockage on dentofacial growth and development are indefinite. [10-11] Several studies have revealed that adenoid hypertrophy and chronic nasal blockage during early childhood period cause dentofacial deformity.[11-12]

Oral respiration causes a higher palatal height.[13] This causes deviations in the posterior septum or it is exaggerated in the existing deviation. Akbay *et al.* detected an association between the height of the palate and posterior deviation of the nasal septum. The deviations had accelerated oral respiration and raised the depth of palatal bone. This phenomenon might increase SD in a blind circle.[8]

This study is designed to determine the effects of septal deviation per se and in combination with concha bullosa on the maxilla and adjacent bones. 

## Materials and Method

In this descriptive-comparative study, 116 samples (71 males and 45 females) were selected from the archive of cone-beam computed tomography (CBCT) images of patients who referred to an oral and maxillofacial radiology clinic in North Iran during 2012–2014. The exclusion criteria were having less than 18 years of age, history of nasoantral mass, fractures of nasomaxillary complex, head and neck syndromes, and orthopedic or orthodontic treatments. All scans were made using a NewTom VG CBCT device (QR s.r.l., Verona, Italy) with a 9-inch field of view (FOV).

**Figure 1:**
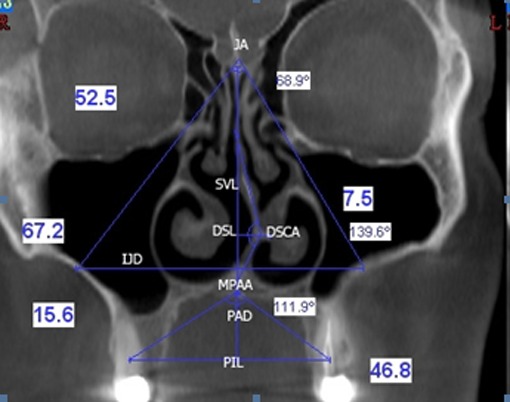
Coronal image of a deviated nasal septum and its measurements

Based on the type of deviations detected, the enrolled samples were divided into four groups (n=29). Group 1 included CBCT images from patients with SD+CB, group 2 were patients with only SD, individuals in group 3 had none of them, and group 4 included patients with only CB. After reconstruction of the coronal images with a 2-mm thickness and intervals based on volumetric image, nominated slices were selected. In these slices, the normal landmarks of crista galli were best detected. The intersection of this perpendicular line passing through crista galli to the palatal bone was considered as the reference point "P". Other measurements were taken with respect to the pre-determined reference points. Followings are the definition of the angle and length based on the selected coronal image (some of these definitions and terms were extracted from Akbey *et al.*’s study[8]):

- Palatal Interalveolar Length (PIL): the distance between the mid-centers of cervical portion of the available tooth structure, from one side to the other. If there is no tooth, then the mid-center of the alveolar bone near the crest is considered as the reference point.
- Palatal Arch Depth (PAD): the length of the line from "P" to the interalveolar line.
- Maxillopalatal Arch Angle (MPAA): the angle that is formed by the lines from "P" to both points of the mid-center of dental or maxillary alveolar bone.
- Septal Vertical Length (SVL): the vertical distance of septum drawn from crista galli to "P".
- Deviated Septal Length (DSL): the length of the horizontal line from the highest point of deviated septum to the vertical axis where the septum is supposed to be.
- Deviated Septal Curve Angle (DSCA): the highest point of the deviated septum taken as an edge, the obtuse angle drawn from this edge point to the crista galli (superior) to "P" (inferior).
- Palatal Arch Depth/ Palatal Inter-alveolar Length (PAD/PIL): the ratio of hard palate depth to interalveolar length.
- Interjugum Distance (IJD): top-view length of the line from the deepest point of a side of the zygomatic arch to the same point on the other side.
- Jugum Angle (JA): the angle that is formed by the lines from crista galli to jugum as the deepest point of zygomatic arch on the right and left sides. (Figure 1)

Table 1 summarizes the abbreviations of the above- used parameters. In this study, when DSCA was less than 150°, the septum was defined as a deviated form.

All data for this study were subjected to statistical analysis using SPSS (ver. 21; SPSS, Chicago, IL). ANOVA was used for overall comparison among the groups and Tukey’s HSD for pairwise comparison. Chi-square test was employed to compare the gender-based variables. The level of statistical significance was established at *p*≤ 0.05.

**Table 1:** Summery of abbreviations

**Abbreviations**	**Extended forms**
CB	Concha bullosa
SD	Septal deviation
PIL	Palatal interalveolar length
PAD	Palatal arch depth
MPAA	Maxillopalatal arch angle
SVL	Septal vertical length
DSL	Deviated septal length
DSCA	Deviated septal curve angle
PAD/PIL	Palatal arch depth/ Palatal inter-alveolar length
IJD	Interjugum distance
JA	Jugum angle

## Results

This descriptive study was performed on four groups of 29 cases to investigate the effects of SD per se and in combination with CB on the depth of the posterior palatal arch. The mean±SD age of patients in all groups was 36.59±11.86. The frequency of males and females were 61.2% and 38.8%, respectively. There were no significant difference between the gender frequency (*p*= 0.64). Table 2 reveals the frequency of female and male individuals as well as the average age of the cases in each group. Based on the results of ANOVA, there was no statistically significant difference among the groups in terms of age (*p*= 0.46). The four groups were significantly different regarding the DSL and DSCA (*p*= 0.0001) (Table 3). The aforementioned findings confirm that the four groups were matched according to the mean age and gender frequency; however, they were differently established concerning the septal deviation.

Table 3 shows the measured values of different variables such as PAD, PIL, SVL, IJD, JA, MPAA, and PAD/PIL ratio for each group. Table 4 displays that no statistically significant differences were determined for the measured variables among the groups and between each pair of groups (*p*> 0.05).

The measured variables had a normal distribution pattern according to a one-sample Kolmogorov-Smirnov test. Thus, to determine the correlation between the main variables (DSCA and DSL) with other parameters in groups 1 and 2, a Pearson's correlation coefficient was used. In group 1, PAD/PIL ratio had a negative correlation with DSCA (r=-0.388, *p*= 0.037). However, a positive correlation was found between PAD/PIL ratio and DSL (r=0.379, *p*= 0.043) (Table 5).

**Table 2:** The frequency of female and male individuals and the mean±SD age of the cases in each group

**Groups**	**Gender**						**Age**	
	**Male**		**Female**		**Total**		**Mea Age**	**Standard Deviation**
	**N**	**%**	**N**	**%**	**N**	**%**		
(1) SD per se	20	69	9	31	29	100	34.14	10.24
(2) SD+CB	18	62.1	11	37.9	29	100	39.14	12.53
(3) Neither SD nor CB	15	51.7	14	48.3	29	100	36.83	13.51
(4) CB per se	18	62.1	11	37.9	29	100	36.24	10.99
Total	71	61.2	45	38.8	116	100	36.59	11.86

**Table 3:** Comparison of conditional variables of DSL and DSCA to confirm differences among the groups

		**N**	**Mean**	**Std. Deviation**	**P**
DSL	1	29	6.20	1.94	0.0001
	2	29	5.62	2.37	
	3	29	0.00	0.00	
	4	29	0.00	0.00	
DSCA	1	29	148	9.63	0.0001
	2	29	152	10.70	
	3	29	180	0.00	
	4	29	180	0.00	

**Table 4:** The mean±SD of measured data in the four groups and each pairwise comparison

	**Group**	**N**	**Mean**	**Std. ** **Deviation**	**P**
PIL (mm)	1	29	41.38	5.56	overall: 0.319 1,2: 0.309 1,3: 0.458 1,4: 0.813 2,3: 0.993 2,4: 0.829 3,4: 0.936
	2	29	43.40	4.13	
	3	29	43.08	3.64	
	4	29	42.41	4.04	
MPAA (°)	1	29	121.06	16.65	overall: 0.613 1,2: 0.988 1,3: 0.801 1,4: 0.999 2,3: 0.606 2,4: 0.998 3,4: 0.716
	2	29	122.28	13.18	
	3	29	117.67	10.50	
	4	29	121.62	15.73	
PAD (mm)	1	29	11.52	3.49	Overall: 0.457 1,2 : 0.976 1,3 : 0.517 1,4: 0.601 2,3: 0.771 2,4: 0.841 3,4: 0.999
	2	29	11.98	3.12	
	3	29	13.05	2.82	
	4	29	12.90	6.49	
PAD/PIL Ratio	1	29	0.28	0.09	overall: 0.639 1,2: 0.997 1,3: 0.833 1,4: 0.862 2,3: 0.729 2,4: 0.765 3,4: 1.000
	2	29	0.27	0.07	
	3	29	0.30	0.05	
	4	29	0.30	0.13	
SVL (mm)	1	29	49.78	5.31	overall: 0.433 1,2: 0.973 1,3: 0.554 1,4: 0.996 2,3: 0.811 2,4: 0.917 3,4: 0.420
	2	29	49.25	4.75	
	3	29	48.15	48.15	
	4	29	50.05	4.66	
IJD (mm)	1	29	61.35	7.90	Overall: 0.287 1,2: 0.744 1,3: 0.212 1,4: 0.720 2,3: 0.781 2,4: 1.000 3,4: 0.803
	2	29	63.20	8.02	
	3	29	64.94	5.09	
	4	29	63.28	6.54	
JA (°)	1	29	71.85	7.27	Overall: 0.813 1,2: 0.957 1,3: 0.980 1,4: 0.985 2,3: 0.808 2,4: 0.999 3,4: 0.880
	2	29	70.99	6.44	
	3	29	72.50	5.57	
	4	29	71.24	6.46	

**Table 5:** Correlation of the main variables of DSL and DSCA with other measured data

**Groups**	**Correlations**			
			**Pearson's Correlation**	**P**
First (N=29)	DSL	PAD	0.322	0.088
		PIL	-0.210	0.275
		MPAA	-0.246	0.197
		PAD/PIL	0.379	0.043
		SVL	-0.106	0.584
		IJD	-0.218	0.255
		JA	0.076	0.693
	DSCA	PAD	-0.358	0.056
		PIL	0.163	0.398
		MPAA	0.268	0.160
		PAD/PIL	-0.388	0.037
		SVL	0.277	0.145
		IJD	0.240	0.209
		JA	-0.165	0.392
Second (N=29)	DSL	PAD	-0.063	0.747
		PIL	0.004	0.982
		MPAA	0.086	0.657
		PAD/PIL	-0.059	0.762
		SVL	0.267	0.161
		IJD	0.154	0.425
		JA	0.074	0.703
	DSCA	PAD	0.014	0.943
		PIL	-0.051	0.794
		MPAA	-0.046	0.814
		PAD/PIL	0.018	0.926
		SVL	0.015	0.937
		IJD	-0.178	0.355
		JA	-0.254	0.184

**Figure 2:**
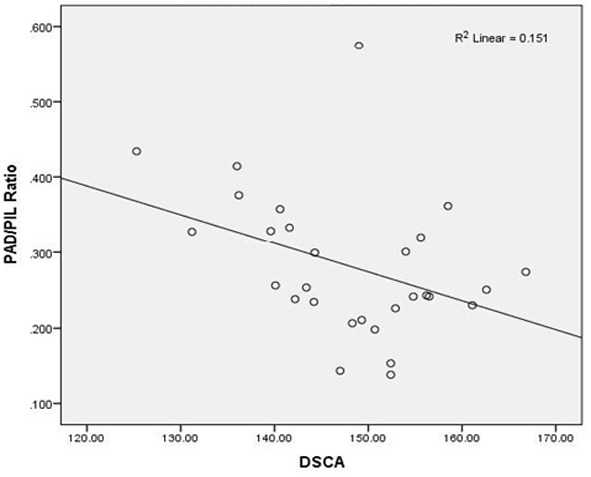
Linear correlation between PAD/PIL ratio and changes in DSCA in group 1

In group 1, a linear correlation coefficient (R^2^=0.151) between the PAD/PIL ratio and DSCA revealed a 15% change in the PAD/PIL ratio as predicted and based on DSCA angle (Figure 2).

## Discussion

SD and CB are the most common anatomical variations. They have been reported variously in different studies.[3] The nasal septum affects the anterior-posterior growth of the maxilla and it was confirmed through studying the homozygous twins.[14] Obstructions of the nasal passage are secondary to SD changes in nasal function and effective for maxillary morphology and structure. It seems that the severity of SD causes partial and complete blockage of the respiratory passage. Narrowing the maxillary arch and the deep palatal arch result from SD. Deep palatal arch is also aggravated by SD.[8]

In this study, none of the measured variables of PAD, PIL, MPAA, PAD/PIL, IJD, JA, SVL, DSL, and DSCA showed statistically significant difference between groups. Akbay *et al.*[8] detected there were significant differences among the groups with different severities of SD. They reported different degrees of SD; whereas in our study, the patients with SD were similar to group I in the study of Akbay *et al.*

The correlation between DSCA and other items in groups 1 and 2 having SD were investigated. DSCA was negatively correlated with PAD/PIL in group 1. This correlation was combined with a decrease in PAD; however, no correlation was detected between DSCA and other parameters in group 1. This indicates that concurrent occurrence of SD and CB remarkably affected the palatal portion of maxilla. Moreover, there was no correlation between DSA and other variables in group 2. Akbay *et al.* observed a negative correlation between PAD/PIL ratio and DSCA angle and a positive correlation between MPAA and DSCA angle.[8]

Evaluation of the correlation between DSL and other items in groups 1 and 2 revealed the DSL to be positively correlated with PAD/PIL only in group 1. This finding was in contrast with Akbay *et al.*’s results[8]since they observed no correlation between DSL and other parameters.

The current study also found no correlation between IJD and JA and the angle of DSCA. Zygomatic bones are the major columns of face that stabilize the face in vertical and horizontal dimensions. These bones are not affected by the nasal septum.[15] Although it is important to determine the type of SD (anterior or posterior), it was not detected in the present study. The posterior type of SD might be the result of caudocranial pressure at the base of septum by raising the palatal bone of the maxilla. This type of deviations is not manipulated in the procedure of rhinoplasty.

In the current study, a pairwise comparison of PAD, PIL, MPAA, PAD/PIL, IJD, JA and SVL was done for all groups. The results showed no statistically significant difference between the parameters.

Drevenšek and Papić[16] found that patients with an incompetent lip had deeper palatal depth rather than those with a competent lip and adequate seal. Berwing *et al.*[1] concluded that mouth breathers had narrower and deeper palates in the posterior portion of palate. Serter *et al.*[10] realized that the depth of maxillary arch was due to the flattened maxillary bone in the group with nasal polyposis. This was lesser than the control group. The results of our study were closer to the findings of Serter *et al.*[10]

In the growth process of the maxillary bone, the palatal bone relocates downward. This relocation is caused by periosteal resorption in the nasal direction and periosteal deposition in the oral direction.[17] Therefore, any obstruction in the nasal airway such as SD and CB could affect the growth and downward remodeling of the palate.

## Conclusion

PAD/PIL ratio had a negative correlation with the DSCA angle. This correlation is combined with a decrease in PAD. These findings suggest that simultaneous occurrence of SD and CB remarkably influences the depth and curve of the base of palate; however, this effect was not observed in the alveolar bone probably due to dental camouflage. 
